# Malaria transmission structure in the Peruvian Amazon through antibody signatures to *Plasmodium vivax*

**DOI:** 10.1371/journal.pntd.0010415

**Published:** 2022-05-09

**Authors:** Jason Rosado, Gabriel Carrasco-Escobar, Oscar Nolasco, Katherine Garro, Hugo Rodriguez-Ferruci, Mitchel Guzman-Guzman, Alejandro Llanos-Cuentas, Joseph M. Vinetz, Narimane Nekkab, Michael T. White, Ivo Mueller, Dionicia Gamboa

**Affiliations:** 1 Unit of Malaria: Parasites and hosts, Institut Pasteur, Paris, France; 2 Sorbonne Université, ED 393, Paris, France; 3 Infectious Disease Epidemiology and Analytics G5 Unit, Institut Pasteur, Paris, France; 4 School of Public Health, University of California San Diego, La Jolla, California, United States of America; 5 Health Innovation Laboratory, Instituto de Medicina Tropical Alexander von Humboldt, Universidad Peruana Cayetano Heredia, Peru; 6 Instituto de Medicina Tropical Alexander von Humboldt, Universidad Peruana Cayetano Heredia, Lima, Peru; 7 Laboratorio de Malaria, Laboratorios de Investigación y Desarrollo, Facultad de Ciencias y Filosofía, Universidad Peruana Cayetano Heredia, Lima, Peru; 8 Universidad Nacional de la Amazonía Peruana, Loreto, Peru; 9 Laboratorio ICEMR-Amazonia, Laboratorios de Investigación y Desarrollo, Facultad de Ciencias y Filosofía, Universidad Peruana Cayetano Heredia, Lima, Peru; 10 Section of Infectious Diseases, Department of Internal Medicine, Yale School of Medicine, New Haven, Connecticut, United States of America; 11 Department of Medical Biology, University of Melbourne, Melbourne, Australia; 12 Population Health and Immunity Division, Walter and Eliza Hall Institute of Medical Research, Melbourne, Australia; 13 Departamento de Ciencias Celulares y Moleculares, Facultad de Ciencias y Filosofía, Universidad Peruana Cayetano Heredia, Lima, Peru; Minia University, EGYPT

## Abstract

**Background:**

The landscape of malaria transmission in the Peruvian Amazon is temporally and spatially heterogeneous, presenting different micro-geographies with particular epidemiologies. Most cases are asymptomatic and escape routine malaria surveillance based on light microscopy (LM). Following the implementation of control programs in this region, new approaches to stratify transmission and direct efforts at an individual and community level are needed. Antibody responses to serological exposure markers (SEM) to *Plasmodium vivax* have proven diagnostic performance to identify people exposed in the previous 9 months.

**Methodology:**

We measured antibody responses against 8 SEM to identify recently exposed people and determine the transmission dynamics of *P*. *vivax* in peri-urban (Iquitos) and riverine (Mazán) communities of Loreto, communities that have seen significant recent reductions in malaria transmission. Socio-demographic, geo-reference, LM and qPCR diagnosis data were collected from two cross-sectional surveys. Spatial and multilevel analyses were implemented to describe the distribution of seropositive cases and the risk factors associated with exposure to *P*. *vivax*.

**Principal findings:**

Low local transmission was detected by qPCR in both Iquitos (5.3%) and Mazán (2.7%); however, seroprevalence indicated a higher level of (past) exposure to *P*. *vivax* in Mazán (56.5%) than Iquitos (38.2%). Age and being male were factors associated with high odds of being seropositive in both sites. Higher antibody levels were found in individuals >15 years old. The persistence of long-lived antibodies in these individuals could overestimate the detection of recent exposure. Antibody levels in younger populations (<15 years old) could be a better indicator of recent exposure to *P*. *vivax*.

**Conclusions:**

The large number of current and past infections detected by SEMs allows for detailed local epidemiological analyses, in contrast to data from qPCR prevalence surveys which did not produce statistically significant associations. Serological surveillance will be increasingly important in the Peruvian Amazon as malaria transmission is reduced by continued control and elimination efforts.

## Author summary

A challenge for eliminating *Plasmodium vivax* is its relapsing nature that can contribute up to 80% of the *P*. *vivax* cases. The detection of this source of transmission remains crucial for focused interventions in settings attempting to achieve elimination. Antibody responses to serological exposure markers to *P*. *vivax* have been validated to detect recent exposure and likely hypnozoite carriers in low transmission settings. To evaluate the benefits of adding serology to the detection of infection in areas where malaria transmission is rapidly reduced, we measured the prevalence of *P*. *vivax* by qPCR and serology in two sites of the Peruvian Amazon. This study shows that antibody responses to *P*. *vivax* antigens can detect more recently exposed people than molecular testing, providing more statistical power for surveillance and critical additional epidemiological knowledge for regions attempting to eliminate malaria transmission.

## Introduction

Peru is considered a low-endemic transmission setting for malaria, with 24,000 cases reported in 2019 [1]. Most of the malaria cases in Peru are concentrated in the northeastern Department of Loreto, in the Amazon basin (96% of cases in 2020) [2]. Even within Loreto, transmission is heterogeneous, i.e., there are spatial and temporal transmission hot-spots surrounded by extensive areas with little or no local transmission. This variation in local malaria transmission is determined by environmental factors such as human settlements close to water bodies [3] and the creation of mosquitoes breeding sites (such as fish ponds, road constructions and mining pools) by deforestation and economic activities [4], as well as occupational factors such as forest-related work [5] or high human mobility between communities [6].

Over the last 20 years, the case incidence of malaria in Peru has fluctuated, influenced largely by the prevailing intervention programs. After the cessation of the Global Fund Malaria Project “PAMAFRO” in 2010, *t*here was a noteworthy increase in the number of malaria cases [7], with a steady rise from 25 005 cases in 2011 to 66 609 cases in 2015. Since then, numbers are descending again due to the Malaria Zero program implemented in 2017 by the Peruvian Minister of Health (MINSA) [8]. In three years of running Malaria Zero program, the number of malaria cases in Loreto decreased by 75% between 2017 and 2020 [9]. In its first phase, this program has strengthened active case detection and early treatment activities involving mainly health promoters from the communities; prevention activities in the population by distributing long-lasting insecticide-treated nets and indoor residual spraying from time to time [10].

*Plasmodium vivax* causes the majority of malaria cases in Peru. The detection of cases is based on light microscopy (LM) diagnosis; this may substantially underestimate prevalence as greater than 50% of infections that are detectable by qPCR may be missed by microscopy [6]. Being based on the detection of blood-stage parasites, LM and qPCR do not detect individuals with only liver-stage hypnozoite infections. Therefore, detecting transmission changes using LM or qPCR data may overlook P. vivax ultra-low-density infections [11] and hypnozoite carriers, thus underestimating transmission levels.

New uses of malaria serological tools have been proposed to inform the impact of such control measures [12]. Ideally, the detection of short-lived antibody responses in a given population’s inhabitants will indicate that recent exposure has happened. Recently, new serological markers of exposure to *P*. *vivax* have been developed and validated for their use in low transmission settings [13]. These serological markers of exposure were able to detect recent exposure in participants from longitudinal cohorts of road and riverine communities with moderate-to-high transmission of the Peruvian Amazon, although performance was notably lower than in low endemic settings [14].

Given that the Malaria Zero program has been implemented in the Peruvian Amazon region, new tools are needed to capture transmission patterns, detecting hot-spots and individuals likely contributing to transmission. In 2018, cross-sectional surveys were executed in two contrasting geographical areas of Loreto: Iquitos (peri-urban communities) and Mazán (rural, riverine communities) to determine the exposure level to *P*. *vivax* through antibody responses to serological markers in the Peruvian Amazon. Whereas previous studies in the Peruvian Amazon only focused on the analyses of antibodies to individual protein such as PvAMA1 [3], PvMSP1_19_ [3], PvMSP8 [15], or PvMSP10 [6], the advantage of our study is the simultaneous measurement of 8 markers of recent exposure to *P*. *vivax*: PvMSP1_19_, PvTRAg_2, PvTRAg_28, PvMSP8, PvMSP3.10, PvRAMA, PvRBP2b and PvEBPII. This provided an excellent opportunity to evaluate the benefits of adding serology to infection detection in monitoring and understanding changes in malaria epidemiology in the context of rapidly intensifying elimination efforts.

## Methods

### a) Ethics statement

Samples were collected from consenting individuals through the CAM study (SIDISI 101645/2017) and the ICEMR study (SIDISI 101518/2018); both studies were approved by the Institutional Ethics Committee of Universidad Peruana Cayetano Heredia (UPCH). UPCH also approved the use of the serum samples collected in those studies and the use of Peruvian negative controls at the Institut Pasteur (SIDISI 100873/2017).

### b) Study area

Two cross-sectional studies were carried out in two districts of Maynas province, Department of Loreto in 2018: Iquitos and Mazán.

Iquitos is the capital of Loreto and is the most populated metropolitan area in the Amazon Region. It is accessed only by air or by river and the majority of the population are mestizo (very mixed-race identity). Three peri-urban communities of the district of Iquitos were surveyed for this study: Rumococha (RC), Santo Tomás (ST), and Quistococha (QC). RC is located 8 km from Iquitos city; ST is 11km from Iquitos city and surrounded by the Nanay River. QC is located 12km from Iquitos city and close to the Nauta-Iquitos Road. The most common occupations are commerce, mototaxi and being a housewife [5].

Mazán is a district of Maynas province located 55–60 km northeast of Iquitos. It is comprised of many small communities, and the community of Mazán is surrounded by the Mazán and the Napo Rivers (3.503° S, 73.094° W). The only access is by speedboat (1 hour from Iquitos). Gamitanacocha (GC), Libertad (LI), Primero de Enero (PE) and Puerto Alegre (PA) are located on the Mazán River. Salvador (SA), Lago Yuracyacu (LY) and Urco Miraño (UM) are located on the Napo River. The main economic activities are farming, timber extraction and fishing [16].

Malaria transmission in Maynas is perennial, with a peak between February and July [17]. While in 2017, Iquitos and Mazán were considered medium- and high-risk districts for malaria transmission, respectively [18], intensified malaria control during 2017 significantly reduced transmission and parasite prevalence in Mazán villages [19].

### c) Study design

In April 2018 (mid transmission season), 933 individuals were enrolled to participate in the Circles of Research on Arboviruses and Malaria (CAM) study executed in the 3 communities of Iquitos mentioned above. In this study, purposive sampling was implemented to the household members presented at the moment of survey. These communities were selected due to their high incidence of *P*. *vivax* according to the MINSA reports of 2017, and due to ease of sample transport to the reference laboratory installed in Iquitos city. The enrolled individuals represented a total of 358 households. From the enrolled participants, 895 individuals consented to provide a blood sample for LM and qPCR diagnosis, and 880 individuals accepted donating a plasma sample for serology purpose (S1 Fig).

In the district of Mazán, a representative population-based cross-sectional survey was conducted in July 2018 (end of transmission season), where 1127 individuals were enrolled to participate in the baseline survey by the Amazonia International Center of Excellence in Malaria Research (ICEMR) team [16]. A total of 261 households were sampled in the 6 communities of Mazán described above. Blood samples were collected for LM and qPCR diagnosis (n = 1127). Plasma samples were collected from 1024 individuals for serological investigation (S1 Fig).

A population survey and geo-referencing of households were implemented in communities from both areas, and data were collected through surveys using tablets and Open Data Kit (ODK) [20,21]. Axillary temperature was recorded. All participants provided written informed consent for participation in the study and for future use of samples in studies of antimalarial antibody responses. Parental written consent and written assent were obtained in the case of child participants. Enrolled participants who did not give informed consent to use a blood sample were excluded from the studies. Peruvian healthy donors from Lima (non-endemic city, n = 90) consented to donate plasma for negative control uses.

### d) Microscopy and molecular diagnosis

Whole blood samples (5 mL) were collected by a venous puncture in citrate tubes. Serum was separated by centrifugation and kept at -80°C until processing. Red blood cell pellets were stored at -20°C prior to molecular diagnosis.

Thick and thin blood smears were stained for 10 min with a 10% Giemsa solution. A slide was declared negative if no malaria parasite was found after examining 100 microscopy fields [22]. Slides were examined by a trained technician at the reference laboratory in Iquitos. All positive slides and 10% of negative slides were confirmed with a second reading by a proficient technician at the reference laboratory in Iquitos.

DNA was isolated from parasitized red blood cell pellets using the E.Z.N.A. Blood DNA Mini Kit (Omega Bio-tek, Inc., Norcross, GA, US), according to the manufacturer’s instructions. Subsequent amplification was performed by a real-time quantitative PCR (qPCR) method using PerfeCTa SYBR Green FastMix 1250 (Quanta bio, Beverly, MA, US) as previously described [6].

### e) Antibody measurements

IgG antibody responses to 12 proteins of *P*. *vivax* were measured using a Luminex platform, as described elsewhere [23]. The panel of serological exposure markers (SEM) to *P*. *vivax* has been previously validated [13] and consisted of 8 proteins: PVX_099980 (19 kDa C-terminal region of merozoite surface protein 1, PvMSP1_19_), PVX_096995 (tryptophan-rich antigen, Pv-fam-a), PVX_112670, PVX_097625 (merozoite surface protein 8, putative, PvMSP8), PVX_097720 (merozoite surface protein 3, PvMSP3.10), PVX_087885 (rhoptry-associated membrane antigen, putative, PvRAMA), PVX_094255 (reticulocyte binding protein 2b, PvRBP2b) and KMZ83376.1d (erythrocyte-binding protein II, PvEBPII). The additionally proteins screened in this study were: PVX_090240 (cysteine-rich protective antigen, putative, PvCyRPA), PVX_110810 (Duffy binding protein, region 2, Sal1 strain, PvDBPSal1), PVX_000930 (sexual stage antigen s16, putative, Pvs16) and PVX_082700 (merozoite surface protein 7 (MSP7), putative, PvMSP7). The complete IgG dataset generated is available on S1 Table.

To account for inter-plate variation, a standard curve was prepared using a plasma pool from hyper-immune adults from Papua New Guinea. Relative Antibody Units (RAU) or dilutions were obtained extrapolating the Median Fluorescence Intensity (MFI) in a standard curve by a 5-parameter logistic model written in R.

### f) Data management and statistics

Demographic data was collected from the databases of the CAM study and the baseline survey of the cohort executed by the Amazonian ICEMR. Categorical covariates were compared using the *χ*^2^ test or Fisher’s exact test when required. Continuous covariates were compared using Mann-Whitney U or *T-* test when required.

Participants were classified as seropositive (i.e., prior exposure in the last 9 months) or seronegative (i.e., no exposure in the last 9 months) to *P*. *vivax* by a seropositivity classifier based on the combined antibody responses to SEM. The seropositivity classifier was a Random Forests based classification algorithm previously validated in low *P*. *vivax* transmission contexts: Thailand (n = 826), Brazil (n = 925), the Solomon Islands (n = 754), and negative controls (n = 274) [13]. The diagnostic target selected was 80% sensitivity and 80% specificity. The algorithm code is available on https://github.com/MWhite-InstitutPasteur/Pvivax_sero_dx.

The proportion seropositive for *P*. *vivax* SEM was estimated as the proportion of individuals classified as seropositive stratified by age. The seropositive rate of individuals per household was estimated to visualise the spatial distribution of seroprevalence to *P*. *vivax*. For the spatial data processing and visualisation, QGIS 2.16 (QGIS Development Team, 2016. QGIS Geographic Information System. Open Source Geospatial Foundation Project) was used.

The association of epidemiological factors with *P*. *vivax* exposure (binary) and *P*. *vivax* parasitaemia (binary) by qPCR were assessed by mixed-effects logistic regression models with random intercepts per households and communities. The following potential risk factors were assessed as fixed effects: age, sex, fever, travels in the last month, outdoor occupation, education level, livestock inside dwelling, indoor spray, and housing type. Factors in final models were selected by manual backward elimination guided by the minimisation of the Akaike information criterion. Three levels were included in these models: individuals within households, households within communities and communities. Intra-class correlation (ICC) was estimated as a measure of correlation and homogeneity at each level [24]. The ICC quantifies the degree of homogeneity of the outcome within clusters. If ICC = 1, there is no within-class variation. If ICC = 0, the observations do not depend on cluster membership. The lme4 R package was utilised for this analysis [25]. Statistical analysis was done using R 3.4.3 (https://www.r-project.org/).

## Results

### a) Socio-demographic characteristics

**Fig 1:**
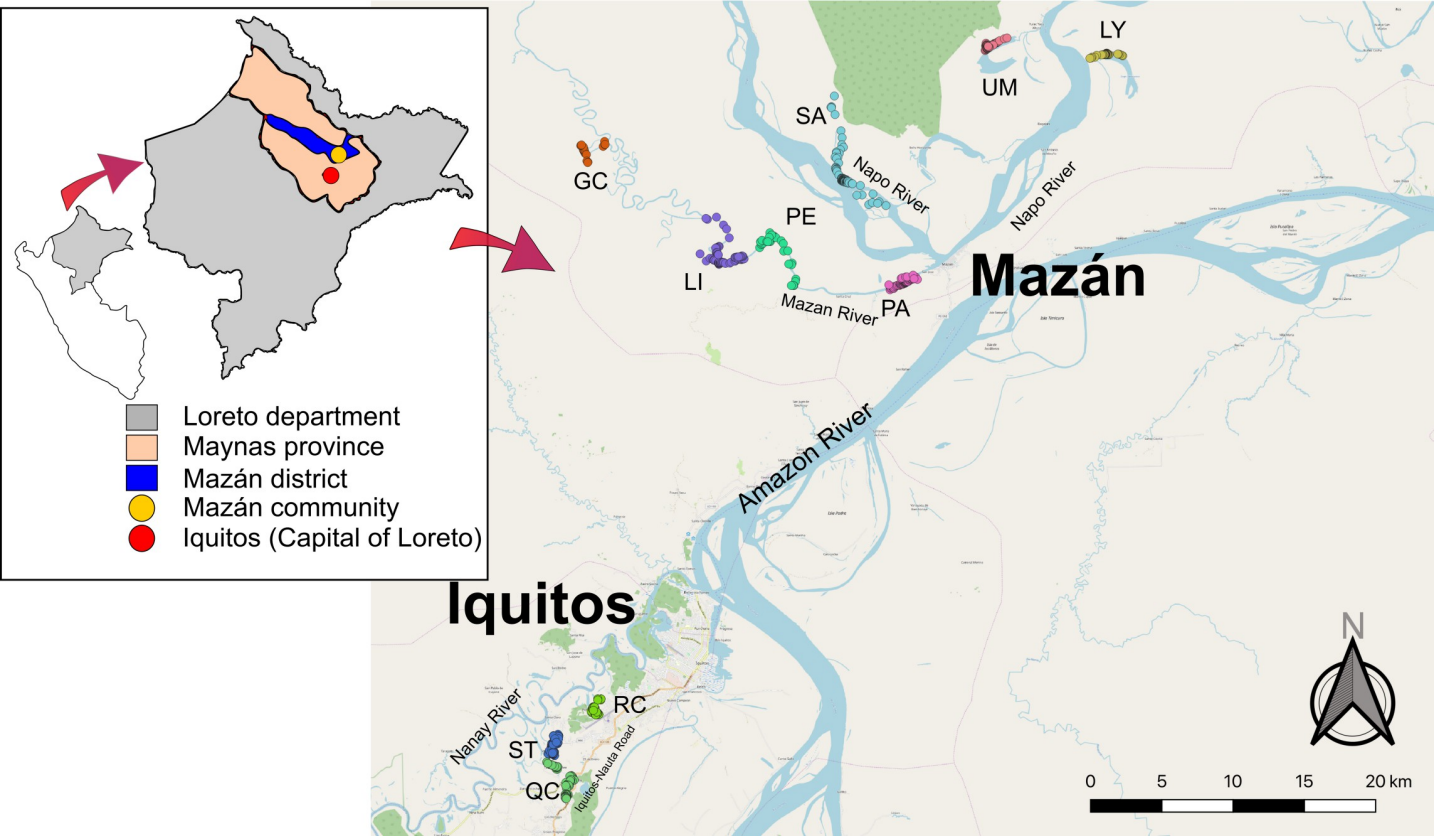
Map of study sites. Mazán and Iquitos are located within the Maynas province, in the department of Loreto. The ICEMR study surveyed seven communities from the Mazán district: Gaminatacocha (GC), Libertad (LI), Primero de Enero (PE), Puerto Alegre (PA), Salvador (SA), Urco Miraño (UM) and Lago Yuracyacu (LY). The CAM study surveyed three communities from Iquitos: Rumococha (RC), Santo Tomás (ST) and Quistococha (QC). Each circle represents a household location. Map generated with QGIS 2.16 (QGIS Development Team, 2016. QGIS Geographic Information System. Open Source Geospatial Foundation Project) based on public geographic data extracted from OpenStreetMap contributors (www.openstreetmap.org) under Open Data Commons Open Database License (ODbL) 1.0 (http://openstreetmap.org/copyright).

Of the 933 surveyed inhabitants in the CAM study, 880 individuals from 358 households (HH) were included in the analysis (Figs 1 and S1). In the CAM study in Iquitos there were 4.70 individuals per HH (95% CI 3.00–6.00) and 52.3% of these HH members were enrolled (2.45 enrolled individuals per HH, 95% CI 2.00–2.89). Of the 1127 surveyed inhabitants in the ICEMR study in Mazán, 1024 individuals from 263 HH were included in the analysis. There were 4.89 individuals per HH (95% CI 3.00–6.00), and 79.6% of HH members were enrolled (3.89 enrolled individuals per HH, 95% CI 3.30–4.47). In the peri-urban area of Iquitos, 35.5% of individuals were under 15 years old, and 59% were women (Table 1). In contrast, in the district of Mazán, 46.3% of individuals were under 15 years old and 51% were women. Almost half of participants of Iquitos had secondary school or higher education level (47.2%), whereas only a fifth of participants of Mazán (18.1%) had this education level (*p* < 0.001). Outdoor occupations such as logger, farmer and fisher were more common in Mazán (40.9%) than in Iquitos (8.9%) (*p* < 0.001). Farmer and housewife were the most common economical occupation in adults older than 18 years old in Mazán (38.1%) and in Iquitos (30.8%), respectively. Only 2.5% of participants from Iquitos and 1.2% from Mazán had fever (axillar temperature > 37.5°C) at the moment of survey (*p* = 0.045).

**Table 1:** Epidemiological characteristics of study sites.

	Iquitos	Mazán	*p*-value
	(n = 880)	(n = 1024)	
**Communities, *n* (%)**			
Rumococha	295 (33.5%)		
Santo Tomás	298 (33.9%)		
Quistococha	287 (32.6%)		
Gamitanacocha		48 (4.7%)	
Libertad		184 (18.0%)	
Primero de Enero		58 (5.7%)	
Salvador		278 (27.1%)	
Lago Yuracyacu		97 (9.5%)	
Puerto Alegre		169 (16.5%)	
Urco Miraño		190 (18.6%)	
**Individual level variables**			
**Age, *median* (min—max)**	26.00 (0–117)	20.00 (0–87)	<0.001
**Age groups, *n* (%)**			
≤ 4.9	56 (6.4%)	127 (12.4%)	<0.001
5–14.9	256 (29.1%)	347 (33.9%)	0.024
15–29.9	199 (22.6%)	134 (13.1%)	<0.001
30–49.9	199 (22.6%)	218 (21.3%)	0.486
≥ 50	170 (19.3%)	198 (19.3%)	0.922
**Gender, *n* (%)**			<0.001
Female	519 (59.0%)	521 (50.9%)	
Male	361 (41.0%)	503 (49.1%)	
**Education**			<0.001
None or primary	462 (52.8%)	749 (81.9%)	
Secondary or higher	413 (47.2%)	165 (18.1%)	
**Outdoor occupation (logger, fisher, and farmer)**			<0.001
Yes	78 (8.9%)	419 (40.9%)	
No	802 (91.1%)	605 (59.1%)	
**Fever, *n* (%)**	22 (2.5%)	12 (1.2%)	0.045
**Household level variables**			
**Households, *n***	358	263	
**Household size**			0.090
< 5 individuals	178 (49.7%)	113 (43.0%)	
≥ 5 individuals	180 (50.3%)	150 (57.0%)	
**Housing type, *n* (%)**			0.004
Open house (0–3 walls)	56 (15.6%)	21 (7.9%)	
Complete	302 (84.4%)	242 (92.1%)	
**Livestock inside dwelling**			0.565
Yes	88 (24.6%)	70 (26.6%)	
No	270 (75.4%)	193 (73.4%)	

Frequency of categorical covariates were compared by χ^2^ test and age median were compared by Mann-Whitney U test. Livestock inside dwelling: Dog, Cat, Chicken.

Crowded households (≥ 5 individuals per HH) were slightly more frequently reported in Mazán than in Iquitos with no significant differences. Open houses have been associated as a high risk factor for *P*. *vivax* in Mazán [16,19] and in other studies in the Amazon [26]. 16% of HH in Iquitos and 8% of HH in Mazán were open houses (0–3 walls) (*p* = 0.004). Having livestock inside dwelling was seen in 24.6% of the HH from Iquitos and 26.6% from Mazán (*p* = 0.565).

### b) Antibody responses signature reveals transmission structure within study areas

*P*. *vivax* prevalence by LM in Iquitos was not significantly different from Mazán (1.8%, 16/880, vs 1.3%, 13/1024, p = 0.329). Although *P*. *vivax* prevalence by qPCR was higher in Iquitos than in Mazán (5.3%, 47/880, vs 2.7%, 28/1024, *p* = 0.005); Mazán (56.5%, 579/1024) showed higher seroprevalence than Iquitos (38.2%, 336/880, *p* < 0.001) (Table2). Only 14% (47/336) and 5% (28/579) of seropositive individuals in Iquitos and Mazán respectively had concurrent, qPCR-detectable infections. There were also significant differences in the antibody responses to 6 out of 8 SEM between the two areas. Antibody responses to PvTRAg_2, PvEBPII, PvRBP2b, PvTRAg_28 and PvMSP3.10 were two times higher in residents from Mazán than Iquitos (*p* mean < 0.001). Participants from Iquitos had 1.2 times higher antibody levels to PvMSP1_19_ than participants from Mazán (*p* = 0.013).

**Table 2:** Prevalence of *P*. *vivax* and *P*. *falciparum*.

	Negative controls	Iquitos	Mazán	*p*-value
	(n = 90)	(n = 880)	(n = 1024)	
**Light microscopy, *n* (%)**				
*P*. *vivax*	0(0%)	16 (1.8%)	13 (1.3%)	0.329
*P*. *falciparum*	0(0%)	3 (0.3%)	5 (0.5%)	0.620
**qPCR, *n* (%)**				
*P*. *vivax*	0(0%)	47 (5.3%)	28 (2.7%)	0.005
*P*. *falciparum*	0(0%)	12 (1.4%)	12 (1.2%)	0.708
**SEM, n (%)**				
*P*. *vivax* seropositive	0(0%)	336 (38.2%)	579 (56.5%)	<0.001
**Antibody levels** * **, mean (95% CI)**				
PvRAMA	0.11 (0.06–0.16)	0.56 (0.43–0.69)	0.65 (0.55–0.74)	0.268
PvTRAg_2	0.10 (0.05–0.15)	0.90 (0.72–1.08)	1.73 (1.50–1.97)	<0.001
PvEBPII	0.02 (0.02–0.03)	0.95 (0.74–1.16)	2.10 (1.83–2.36)	<0.001
PvRBP2b	0.04 (0.03–0.06)	1.44 (1.21–1.66)	3.13 (2.84–3.43)	<0.001
PvMSP1_19_	1.16 (0.69–1.64)	3.58 (3.20–3.93)	2.98 (2.67–3.29)	0.013
PvMSP8	0.03 (0.02–0.03)	0.46 (0.32–0.61)	0.56 (0.43–0.68)	0.343
PvTRAg_28	0.66 (0.37–0.95)	2.09 (1.83–2.35)	3.66 (3.33–3.99)	<0.001
PvMSP3.10	0.05 (0.03–0.06)	0.46 (0.35–0.57)	1.20 (1.02–1.38)	<0.001

Abbreviations: 95% CI = 95% confidence interval. Comparisons were made between participants from Iquitos and Mazán. Healthy donors were included as negative controls

*Antibody level values are in relative antibody units (RAU) interpolated from standard curves using a 5PL logistic regression model. RAU were values going from 0.02 to 0.00000195. RAU values were multiplied by 1000 to ease reading. Frequencies were compared by χ2 test or Fisher’s exact test when required, and antibody levels were compared by *T*-test.

**Fig 2:**
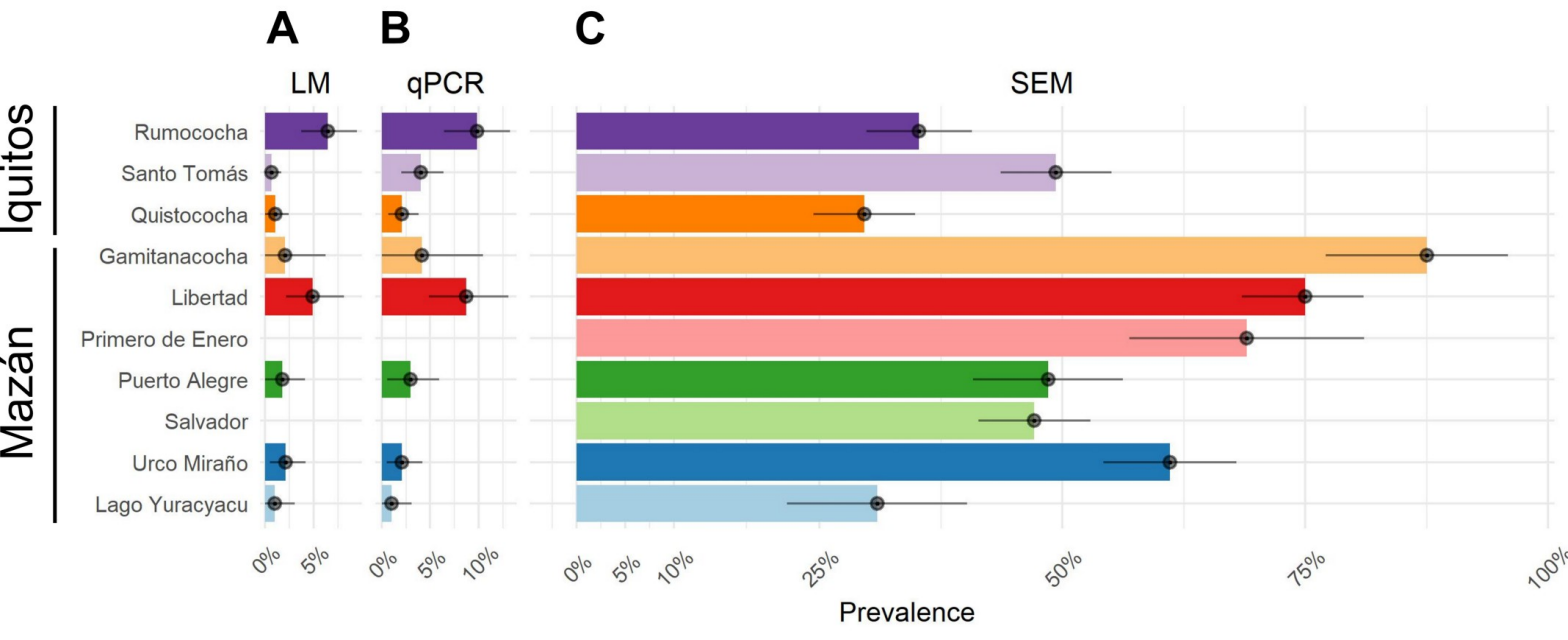
Prevalence of *Plasmodium vivax* measured by three tools in communities of Iquitos and Mazán. **A)** Prevalence of *P*. *vivax* by light microscopy (LM). **B)** Prevalence of *P*. *vivax* by qPCR and **C)** Prevalence of *P*. *vivax* by antibody responses to SEM. Dots denote the mean and lines denote the 95% confidence intervals.

When exploring the prevalence of *P*. *vivax* by community, we found high heterogeneity within study sites (Fig 2). Both LM and qPCR prevalence had similar spatial patterns in both study sites (Fig 2A and 2B). For better interpretation of prevalent infections in this study, we refer only to the qPCR data from now on. In Iquitos, Rumococha showed higher PCR prevalence (9.83%, 29/295) than Santo Tomás (4%, 12/298) (*p* = 0.005) and Quistococha (2%, 6/287) (*p* < 0.001). Conversely, seroprevalence was higher in Santo Tomás (49.3%, 147/298) than Rumococha (35.35%, 104/295) (*p* < 0.001) and Quistococha (29.6%, 85/287) (*p* < 0.001).

In Mazán, Libertad had the highest qPCR prevalence among all the communities (8.7%, 16/184), followed by Gamitanacocha (4.2%, 2/48), Puerto Alegre (2.9%, 5/169), Urco Miraño (2.1%, 4/190), and Lago Yuracyacu (1.0%, 1/97). Salvador and Primero de Enero did not have any positive qPCR sample in the survey (0%). On the other hand, Gamitanacocha was the community with higher seroprevalence (87.5%, 42/48), followed by Libertad with 75% (138/184) of seroprevalence, Primero de Enero (69%, 40/58), Urco Miraño (61.0%, 116/190), Puerto Alegre (48.5%, 82/169), Salvador (47.1%, 131/278) and Lago Yuracyacu (30.9%, 30/97).

### c) Seropositive rate increase with age in both study sites but was more pronounced in Mazán

Antibody levels and seropositivity rate increased with age in both study sites (S3 Fig and S2 and S3 Tables). The proportion of seropositive individuals was significantly higher in participants from Mazán than Iquitos in all age groups (age group < 15: 1.72 times higher; age group 15–29.9: 1.92 higher; age group 30–49.9: 1.54 higher; age group 50+: 1.39 higher; *p* mean < 0.001) (S3 Fig). There was a notable increase in the proportion seropositive people aged > 15 years in both study sites (S3 Fig). This difference was more pronounced in Mazán, where the SEM seropositivity rate in the age group 15–29.9 (0.69, 95% CI 0.60–0.76) was two times higher than in the age group 0–15 (0.31, 95% CI 0.27–0.35) (*p* = 0.006) (S3 Fig). Antibody titers to individual SEM also showed an age-trend in both sites for all antigens considered (S4 Fig).

**Fig 3:**
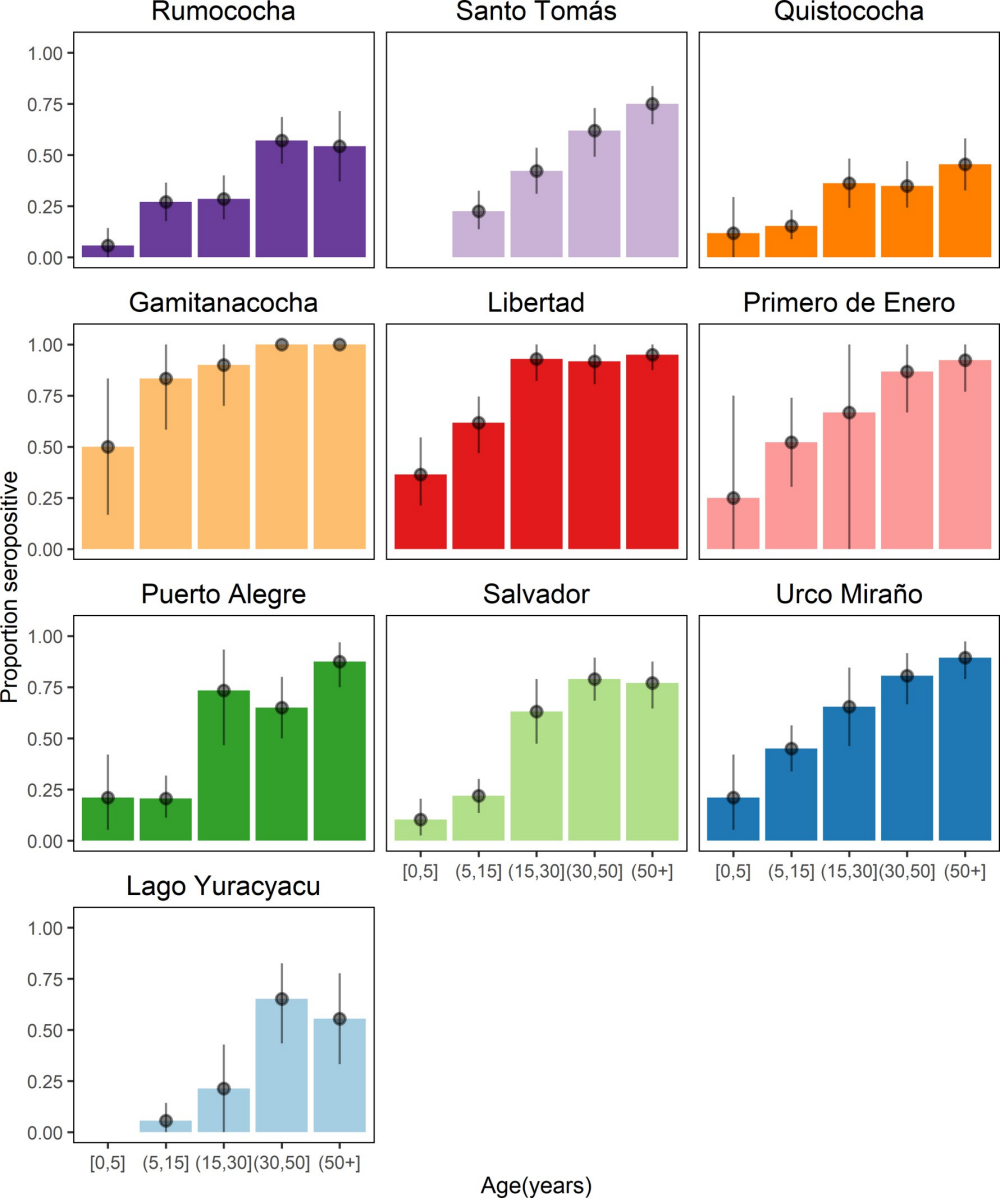
Age trend of seropositivity to *P*. *vivax* in the studied communities. Each panel indicates the age seroprevalence detected by antibody responses to SEM in the villages of Iquitos: Rumococha, Santo Tomás and Quistococha, and the villages of Mazán: Gaminatacocha, Libertad, Primero de Enero, Puerto Alegre, Salvador, Urco Miraño and Lago Yuracyacu. Dots denote the proportion mean and 95% confidence interval of seroprevalence per age-group.

Within each study area, villages had different seropositive rate-age patterns (Fig 3). The lowest seropositive rates in participants aged <15 years were observed in Lago Yuracyacu (0.04, 95% CI 0.00–0.11) in Mazán, and Quistococha (0.15, 95% CI 0.08–0.22) in Iquitos. The highest seropositive rates in participants aged <15 years were observed in Gamitanacocha (0.72, 95% CI 0.50–0.88) in Mazán, and Santo Tomás (0.21, 95% CI 0.13–0.31) in Iquitos. Lago Yuracyacu (0.21, 95% CI 0.00–0.43) in Mazán and Rumococha (0.28, 95% CI 0.18–0.40) in Iquitos had the lowest seropositive rate in participants aged 15–29.9 years, respectively. Seropositivity rate was 0.93 in participants aged 15–29.9 years from the village of Libertad (0.93, 95% CI 0.82–1.00), and 0.42 in Santo Tomás (0.42, 95% CI 0.31–0.53) (Fig 3).

### d) Stratification of transmission was detected by seropositivity maps

**Fig 4:**
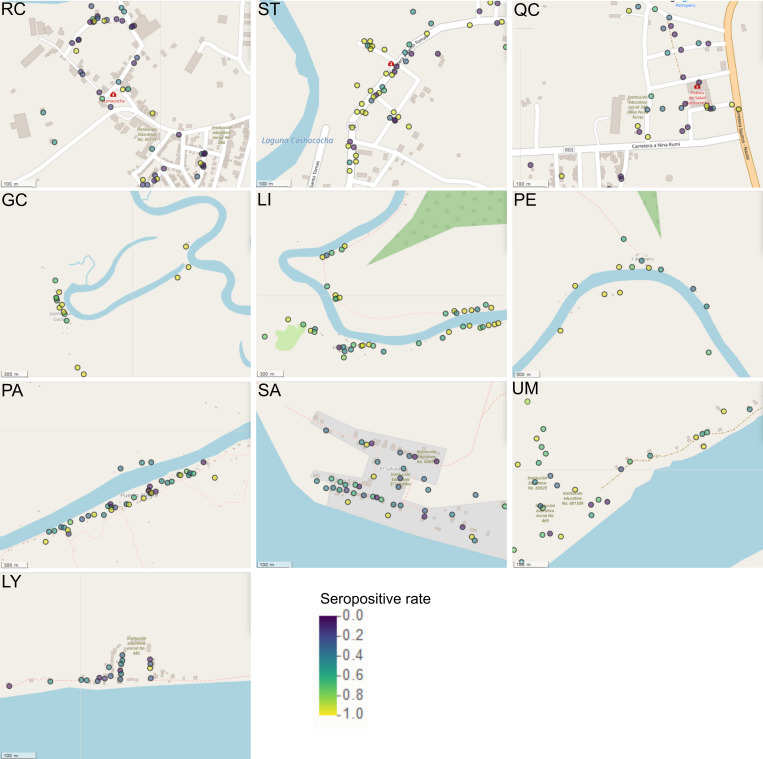
Spatial distribution of seropositive cases in the study sites. The seropositive rate per household was calculated as the proportion of seropositive individuals per number of household members participating in the study. The figure depicts households in the villages of Iquitos: Rumococha (RC), Santo Tomás (ST) and Quistococha (QC), and the villages of Mazán: Gaminatacocha (GC), Libertad (LI), Primero de Enero (PE), Puerto Alegre (PA), Salvador (SA), Urco Miraño (UM), and Lago Yuracyacu (LY). Each point indicates a household location. Households are colored accordingly to their seropositive rate. Map generated with QGIS 2.16 (QGIS Development Team, 2016. QGIS Geographic Information System. Open Source Geospatial Foundation Project) based on public geographic data extracted from OpenStreetMap contributors (www.openstreetmap.org) under Open Data Commons Open Database License (ODbL) 1.0 (http://openstreetmap.org/copyright).

In order to detect spatial patterns of exposure within communities, a seropositivity rate per household was estimated for both areas. The household-level spatial distribution of individuals is represented in Fig 4.

In the peri-urban areas of Iquitos, Santo Tomás had the highest proportion of households with seropositive rate ≥ 0.75 (40%), in contrast to Rumococha (20%) and Quistococha (20%) (S5 Fig). However, the distribution of households with high positive rate did not show spatial structure, i.e., there were households with ≥ 0.75 seropositive rate surrounded by households with < 0.5 seropositivity rate, indicating thus an unstable transmission in Rumococha and Quistococha.

A more heterogeneous transmission landscape was found in the communities of Mazán, probably related to the geographical location of households. Mazán district is crossed by two rivers: Mazán River and Napo River. The highest proportions of seropositive individuals at household level were detected in communities along the basin of Mazán River: Gamitanacocha (88%), Libertad (63%) and Primero de Enero (60%). Although also located in the basin of Mazán River, only 31% of the households in Puerto Alegre had ≥ 0.75 seropositive rate. While the proportion of households with high seropositive rate in the communities of the basin of Napo River were the following: Urco Miraño (45%), Salvador (20%) and Lago Yuracyacu (4%) (S5 Fig). The exposure map in Mazán indicates that transmission is stable and more frequent in households rounding the Mazán River than the Napo River.

### e) Age and being male were associated with seropositivity in the rural and in the peri-urban areas

**Fig 5:**
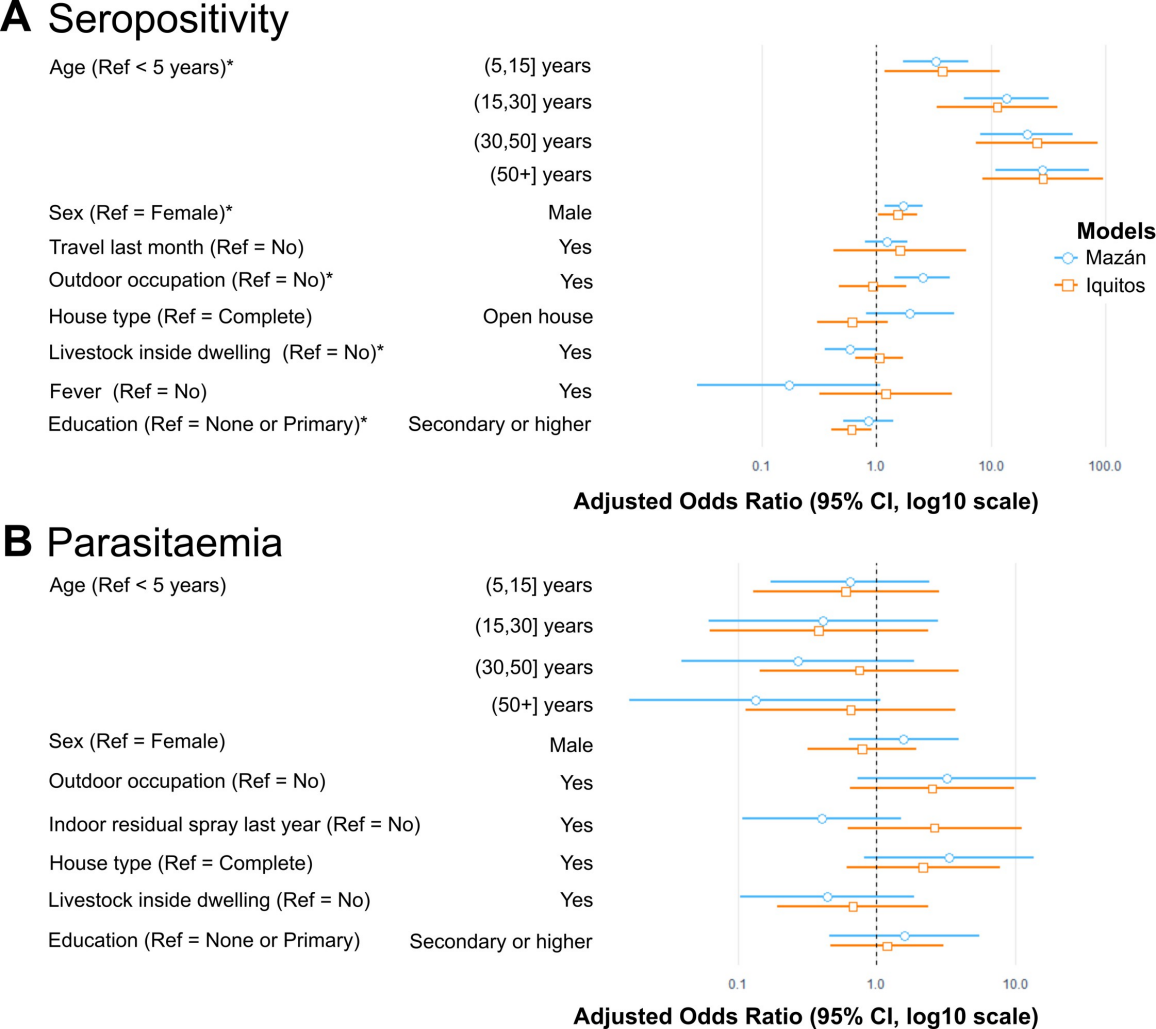
Visual summary of estimates of multilevel logistic regression models of *P*. *vivax* seropositivity and parasitaemia in Mazán and Iquitos. A) Fixed effects for seropositivity to *P*. *vivax*. B) Fixed effects for *P*. *vivax* parasitaemia; no factors were significantly associated. Adjusted Odds Ratios and the 95% CI are represented by blue (Mazán) and orange (Iquitos) lines. Ref = Reference; *: *p* < 0.05.

Due to the multilevel data structure of this study, we implemented multilevel multi-community logistic regression models at three levels: individual, households, and communities. The exposure output was measured by seropositivity to SEM, whereas the *P*. *vivax* parasitaemia output was measured by PCR positivity. The effect of households and community levels on individual outcomes were measured by the intra-class correlation (ICC). The ICC quantifies the size of the general contextual effect, so the more relevant the context is for understanding individual differences in the outcome, the higher the ICC [27]. The estimates of the random effects coefficients for null and the multilevel multi-community models are summarised in S4 and S6 Tables. The ICC values are presented in S5 and S7 Tables. The significant fixed effects for the multilevel multi-community models are graphically represented in Fig 5.

In Mazán, the risk of being seropositive to *P*. *vivax* increased with increasing age (adjusted odds ratio, aOR_5-14.9 years_ = 3.27; 95% CI 1.69–6.31; *p* < 0.001; aOR_15-29 years_ = 13.68; 95% CI 5.78–32.38; *p* < 0.001; aOR_30-49.9 years_ = 20.58; 95% CI 8.18–51.79; *p* < 0.001; aOR_≥50 years_ = 28.34; 95% CI 11.05–72.68; *p* < 0.001), being male (aOR = 1.72; 95% CI 1.17–2.51; *p* = 0.01) and working outside (aOR = 2.51; 95% CI 1.43–4.41; *p* < 0.001). Having domestic animals was associated with a low risk of being seropositive (aOR = 0.59; 95% CI 0.35–0. 98; *p* = 0.04). There was no association with fever symptom and seropositivity, as well with the travel history in the last month and the type of house (S4 Table). The high ICC value for community, in contrast to households, indicated that seropositivity of participants was mostly affected by community factors or that there was homogeneity within communities, e.g., closeness to water bodies. This possibly indicates a more uniform and stable local transmission pattern (S5 Table).

In the peri-urban area of Iquitos, the risk to *P*. *vivax* seropositivity increased with increasing age (aOR_5-14.9 years_ = 3.76; 95% CI 1.17–12.02; *p* 0.03; aOR_15-29 years_ = 11.35; 95% CI 3.35–38.45; *p* < 0.001; aOR_30-49.9 years_ = 25.18; 95% CI 7.43–85.37; *p* < 0.001; aOR_≥50 years_ = 28.51; 95% CI 8.46–96.05; *p* < 0.001). There was an association between being male and being seropositive to *P*. *vivax* (aOR = 1.52; 95% CI 1.02–2.26; *p* = 0.04). Having a high education level (either secondary school or higher) was a protective factor (aOR = 0.60; 95% CI 0.40–0.91; *p* = 0.02). History of travel in the past month was not associated with seropositivity. The ICC values indicated that the household variable explains considerably more variance of seropositivity to *P*. *vivax*. This possibly indicates a heterogeneous individual exposure that could be related to job- related habits rather than to community factors (S5 Table).

No factors were significantly associated with high or low risk of parasitaemia in Mazán and Iquitos (S6 Table). On the other hand, the ICC showed that households within community and community levels contributed equally to the variance for *P*. *vivax* parasitaemia in Mazán. In contrast, households within the community explained more of the variance for *P*. *vivax* parasitaemia in Iquitos (S7 Table).

## Discussion

Here we show that IgG antibody responses to 8 *Plasmodium vivax* recombinant proteins used as serological exposure markers (SEM) identified more exposures (both current and previous) than molecular testing in two low transmission regions of the Peruvian Amazon. The measures of *P*. *vivax* seroprevalence resulted in greater statistical power for epidemiological analyses than qPCR parasite prevalence in such settings with low transmission rates. *P*. *vivax* SEM will be a key tool for serological surveillance as malaria transmission is reduced by continued control and elimination efforts in the Peruvian Amazon.

We used a Random Forest algorithm that incorporated the combined antibody responses to the 8 SEM to classify surveyed individuals into seropositive or seronegative, i.e., recent exposure to *P*. *vivax* in the last 9 months. The seroprevalence of *P*. *vivax* was higher in the riverine communities of Mazán than in the peri-urban communities of Iquitos. Seroprevalence increased with age in both sites, with a significant increase in participants older than 15 years old, although the distribution by age was more heterogeneous among communities from Mazán. The distribution of seropositive cases did not show a spatial structure in communities of Iquitos but did it in Mazán.

Age and being male were factors associated with high *P*. *vivax* seropositivity in both areas, while having higher education level and livestock inside dwelling were low-risk factors in Iquitos and Mazán, respectively. Age is a factor that can be linked to i) lifetime cumulative exposure (i.e., adults living all their lifetime in an endemic area would have been more exposed to *P*. *vivax* than children or newcomers) or ii) labor exposure (i.e., people in adult age increase their chances of exposure by working in forest-related jobs). Our previous work showed that antibody levels increased with the recency of infection, the number of blood-stage infections and age [14]. However, age is the stronger predictor for antibody levels in communities with high transmission level [14]. The presence of livestock in the households has been associated with low *P*. *vivax* prevalence in the community of Urco Miraño [16]. Previous reports in these communities show that mosquito’s blood meal preferences are human, followed by cow and Galliformes [28]. This opportunistic feeding behaviour depends on the host availability during the biting times at night [29]. From our study, we could hypothesise that the protective effect of having domestic animals is the reflection of the host availability during the biting time in some communities in Mazán; however, more data is needed to understand this phenomenon. Moreover, outdoor occupation was a risk factor for *P*. *vivax* seropositivity in Mazán only, which is in line with previous reports of the malaria epidemiology in the area [5].

The high seroprevalence in Iquitos and Mazán is consistent with recent intense exposure in both sites [5,19,28]. Our serological markers have been validated for detecting recent exposure in low transmission settings [13], but in high transmission settings, seropositivity may be due to either recent infection or cumulative lifetime exposure [14]. This limits the utility of these serological markers as individual-level markers of exposure in high transmission settings. However, high antibody levels in young people could indicate recent exposure, as their lifetime exposure is lower than adults [14]. To better understand recent exposure in these settings, we could rely on the antibody levels in the younger groups.

The choice of tools to detect exposure to *P*. *vivax* could have a significant impact on the control measures implemented by health authorities. For instance, in population screening studies in Mazán, only 23% of the infections were detected by LM-based active case detection, compared to qPCR [30]. Also, of the qPCR positive infections, 57% were asymptomatic [30]. In low transmission settings, where antibodies to SEMs represent exposure in a defined period of 9 months, SEMs outperform qPCR to detect transmission patterns as it is both more sensitive and less susceptible to transmission seasonality than qPCR [12]. In our study, compared to qPCR, SEM detected up to 4 and 11 times more of the <15 years participants recently exposed to *P*. *vivax* in Iquitos and Mazán, respectively. However, our study showed that in areas that after long periods of moderate-to-high transmission rapidly goes to low transmission, antibodies remain at high levels for several years after transmission has been reduced. As a consequence, they are not well suited to measure short-term changes in transmission in areas where populations have established high levels of immunity. This could impact the use of antibodies to measure the impact of interventions [12,31]. We thus recommended that in areas where transmission has shifted from high-moderate to low levels like Iquitos and Mazán, SEMs are best suited to detect recent exposure in young people.

Some limitations of this study are the lack of information about recent malaria episodes and time of residence in the study survey to better explain the communities’ transmission patterns and the relationship with seropositvity. The differences in the timing of execution of each study could have influenced the LM and qPCR prevalence in both sites, i.e., in Iquitos, samples were collected during transmission months. In contrast, in Mazán, samples were collected after the transmission peak. The low proportion of participants sampled per household in Iquitos could have overestimated the seropositive rate per household and thus the differences between households detected by our multilevel logistic model. This sampling bias may have also affected the distribution of seropositive cases or maps of seropositivity.

Overall, this study confirms that malaria transmission in the Peruvian Amazon is heterogeneous and that geographical, socio-demographic and human behaviour are important factors for the maintenance of disease burden in this region. Our findings have implications for designing appropriate interventions against malaria in the region; these novel serological markers could be deployed as a tool to target individual and community interventions, but care needs to be taken with their use in areas where transmission has been reduced rapidly from high to low transmission.

## Supporting information

S1 Fig Flowchart of the study design. CAM: Circles of Research on Arboviruses and Malaria study, ICEMR: Amazonia International Center of Excellence in Malaria Research, HH: Households, Ind: Individuals.
(TIF)

S2 Fig Age distribution of participants enrolled in the study stratified by gender. (TIF)

S3 Fig Age curves of *P*. *vivax* infections and exposure in participants of Iquitos and Mazán stratified by gender. A) Proportion of individuals with ongoing infections detected by Light microscopy (LM). B) Proportion of individuals with ongoing infections detected by qPCR. C) Proportion of individuals exposed to *P*. *vivax* detected by antibody responses to serological exposure markers.
(TIF)

S4 Fig Association between antibody responses to 12 *P*. *vivax* antigens and age. A) Age curve of antibody titers in Iquitos and Mazán. B) Distribution of antibody titers stratified by seropositivity given by the Random Forests based classification algorithm. Note the controls are grouped together regardless of age.
(TIF)

S5 Fig Distribution of seropositive rate per households in the studied communities. Seropositive rate per household was estimated as the proportion of seropositive individuals classified per number of household members participating in the study.
(TIF)

S1 Table Complete dataset (IgG antibody responses and epidemiological variables). (XLSX)

S2 Table Correlation between antibody levels and age. (DOCX)

S3 Table Proportion of participants with *P*. *vivax* infections detected by microscopy, qPCR, and serology. (DOCX)

S4 Table Fixed Effects of multi-community multilevel models for seropositivity to *P*. *vivax*. (DOCX)

S5 Table Random effects of multilevel logistic regression models of seropositivity to *P*. *vivax*. (DOCX)

S6 Table Fixed Effects of multi-community multilevel models for *P*. *vivax* parasitaemia. (DOCX)

S7 Table Random effects of multilevel logistic regression models of *P*. *vivax* parasitaemia. (DOCX)
