# Supplementary figures and images for: Malaria transmission structure in the Peruvian Amazon through antibody signatures to Plasmodium vivax
Source: PLoS Negl Trop Dis. 2022 May 9;16(5):e0010415. doi: 10.1371/journal.pntd.0010415 (PMC9119515; doi:10.1371/journal.pntd.0010415)

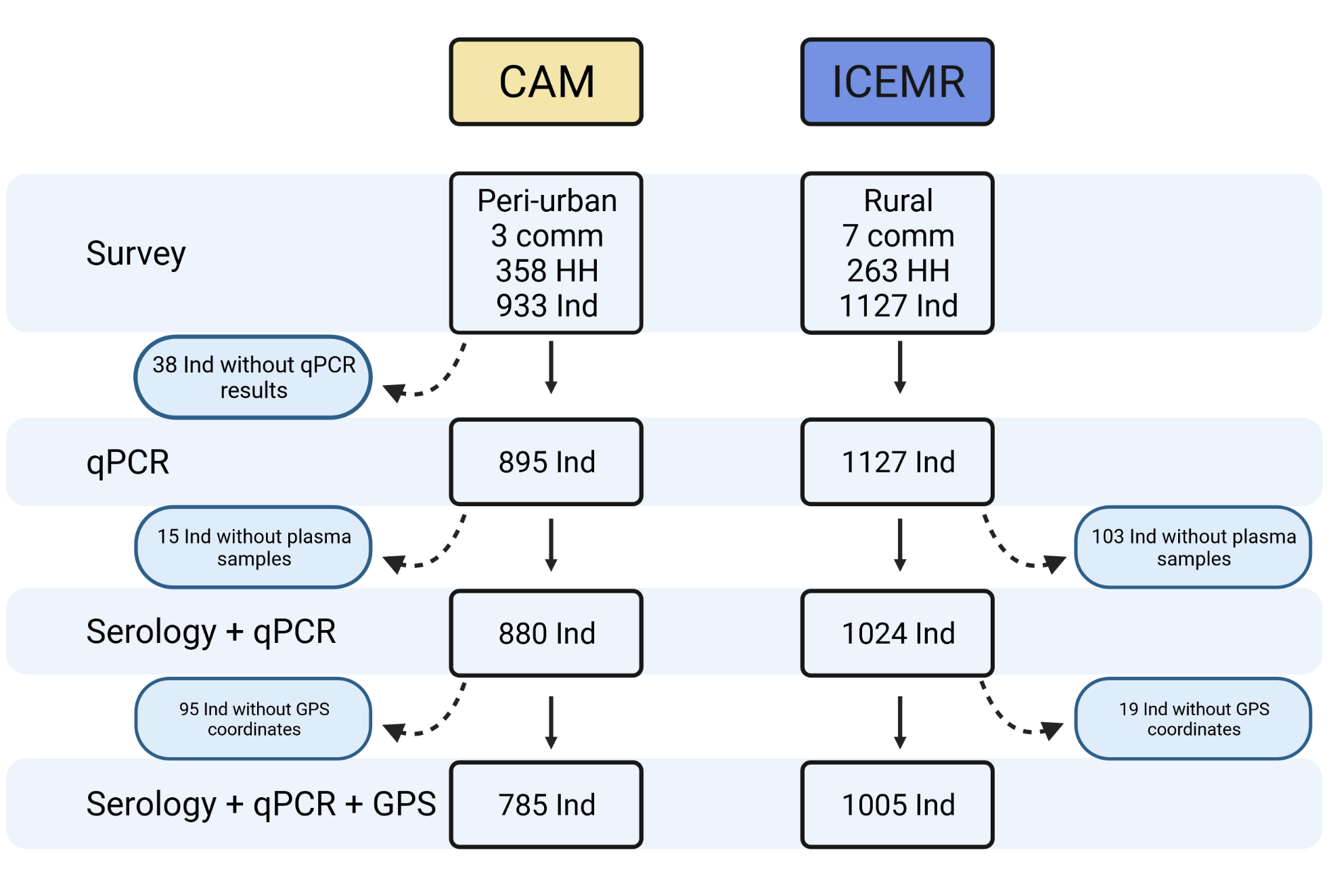

Supplement: S1 Fig — CAM: Circles of Research on Arboviruses and Malaria study, ICEMR: Amazonia International Center of Excellence in Malaria Research, HH: Households, Ind: Individuals. (TIF) [file pntd.0010415.s001.tif]

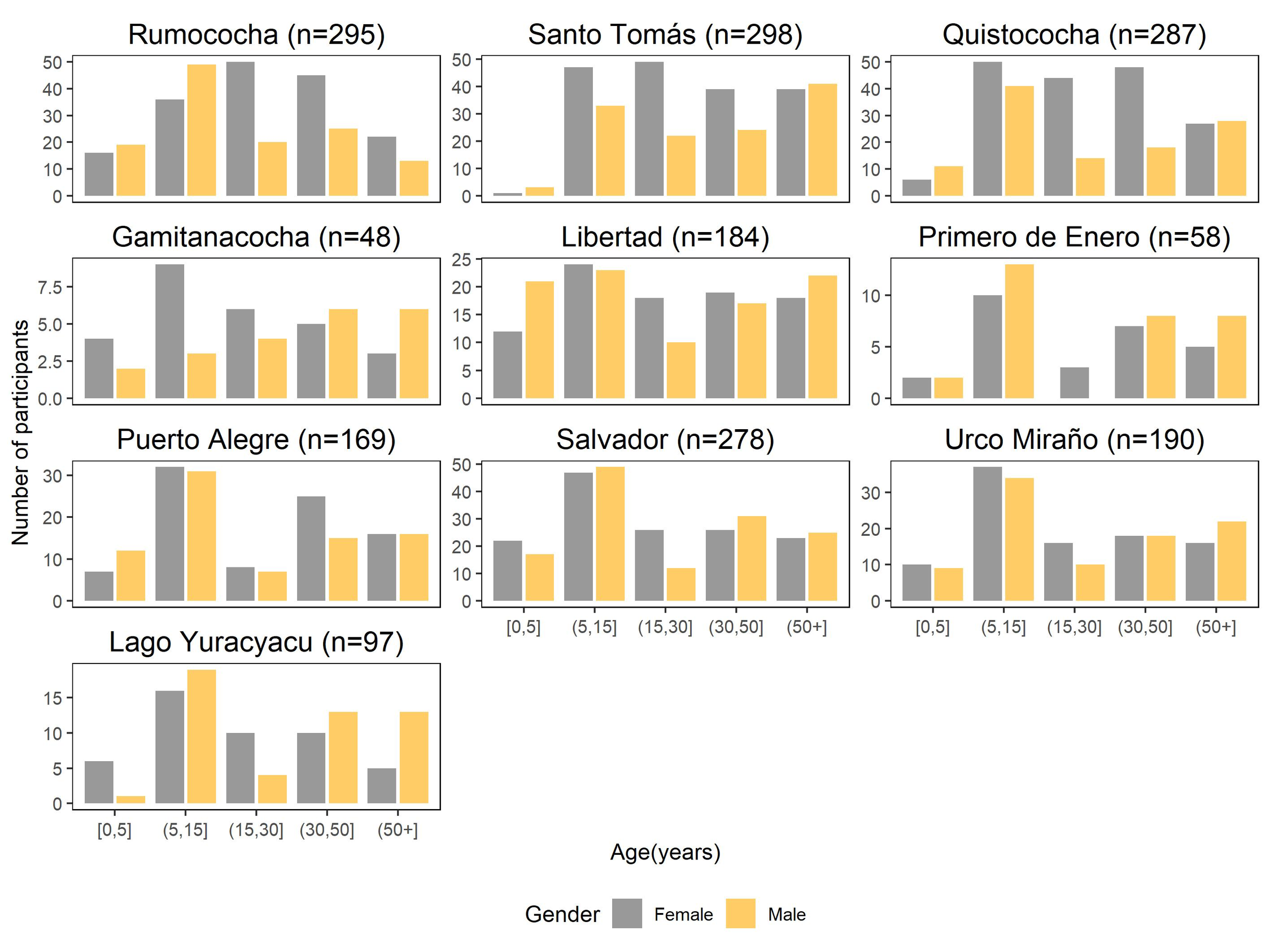

Supplement: S2 Fig — (TIF) [file pntd.0010415.s002.tif]

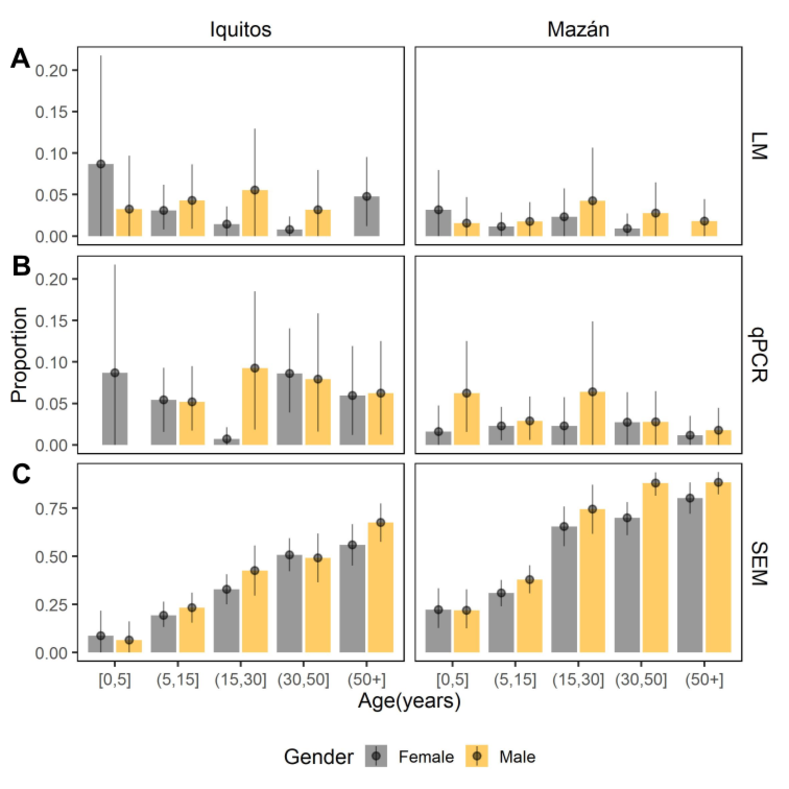

Supplement: S3 Fig — A) Proportion of individuals with ongoing infections detected by Light microscopy (LM). B) Proportion of individuals with ongoing infections detected by qPCR. C) Proportion of individuals exposed to P. vivax detected by antibody responses to serological exposure markers. (TIF) [file pntd.0010415.s003.tif]

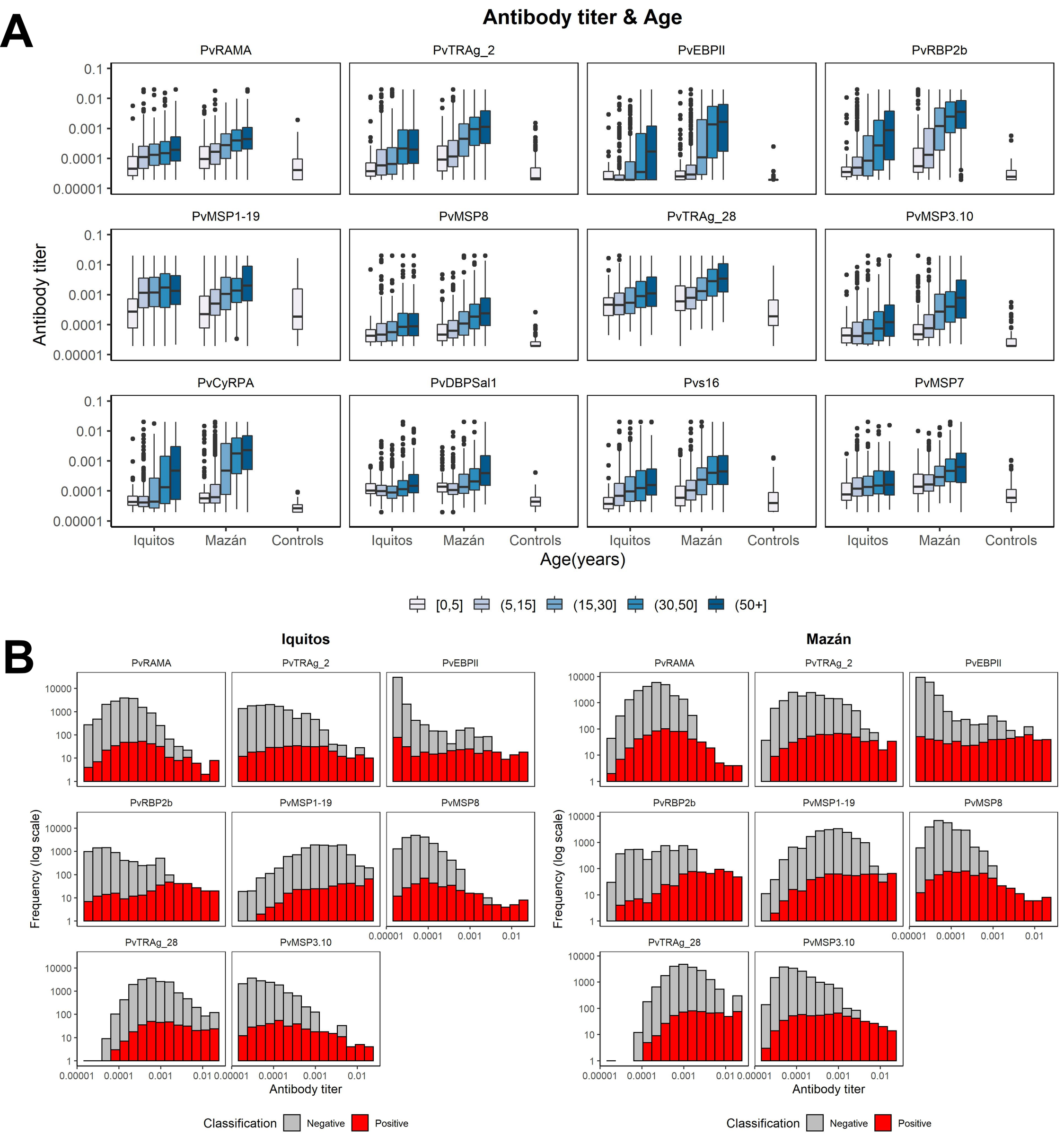

Supplement: S4 Fig — A) Age curve of antibody titers in Iquitos and Mazán. B) Distribution of antibody titers stratified by seropositivity given by the Random Forests based classification algorithm. Note the controls are grouped together regardless of age. (TIF) [file pntd.0010415.s004.tif]

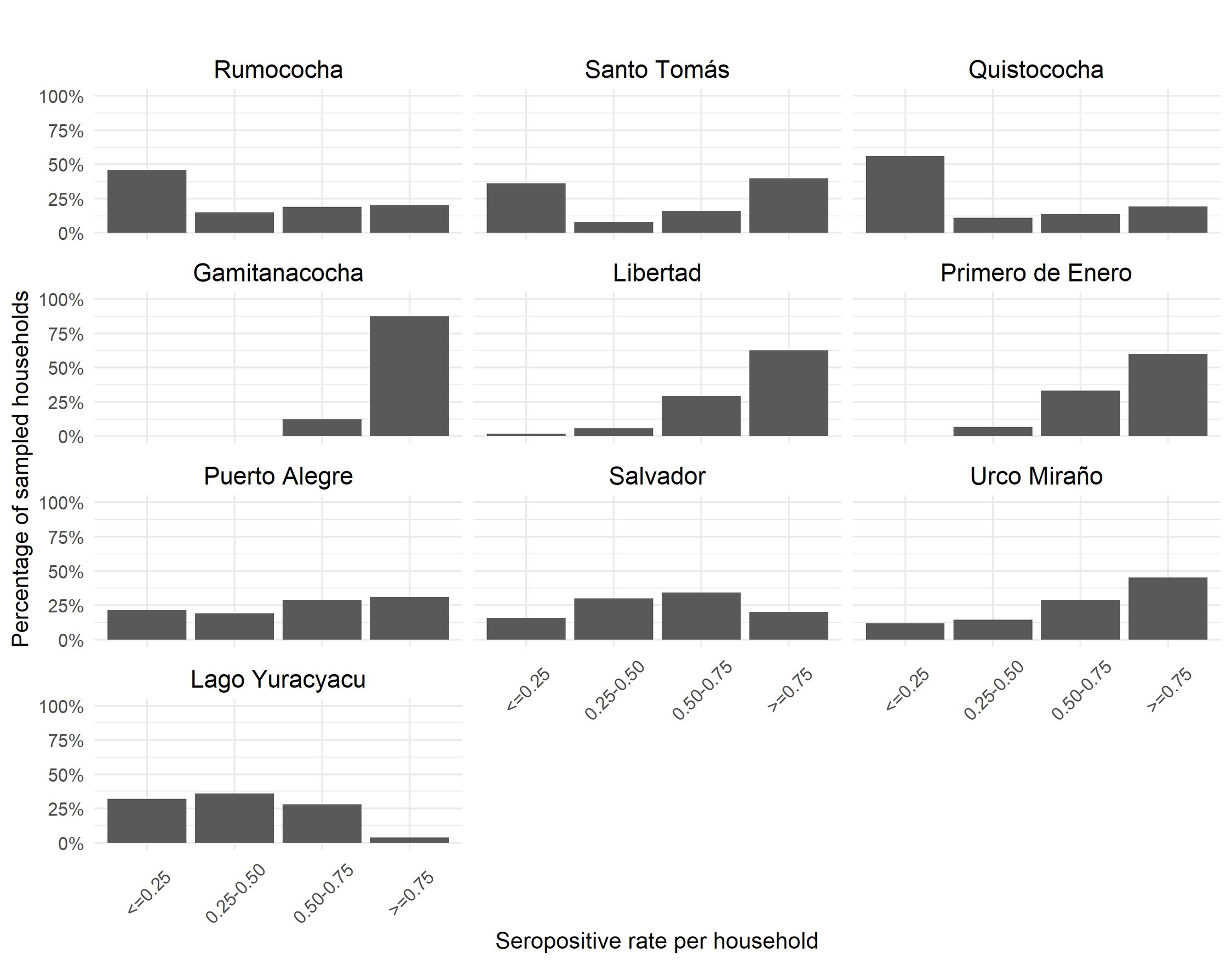

Supplement: S5 Fig — Seropositive rate per household was estimated as the proportion of seropositive individuals classified per number of household members participating in the study. (TIF) [file pntd.0010415.s005.tif]
