# Supplementary material for: Malaria transmission structure in the Peruvian Amazon through antibody signatures to Plasmodium vivax
Source: PLoS Negl Trop Dis. 2022 May 9;16(5):e0010415. doi: 10.1371/journal.pntd.0010415 (PMC9119515; doi:10.1371/journal.pntd.0010415)
Supplement: S2 Table — (DOCX) [file pntd.0010415.s007.docx]

| **S2 Table. Correlation between antibody levels and age.** | | | | |  |
| --- | --- | --- | --- | --- | --- |
| **Antibody response** | **Iquitos** | | **Mazán** | |  |
|  | **Spearman's rho** | ***p*-value** | **Spearman's rho** | ***p*-value** |  |
|  |  |  |  |  |  |
| PvCyRPA | 0.335 | < 0.001 | 0.547 | < 0.001 |  |
| PvRAMA | 0.223 | < 0.001 | 0.401 | < 0.001 |  |
| PvTRAg_2 | 0.301 | < 0.001 | 0.485 | < 0.001 |  |
| PvEBPII | 0.326 | < 0.001 | 0.557 | < 0.001 |  |
| PvDBPSal1 | 0.149 | < 0.001 | 0.347 | < 0.001 |  |
| PvRBP2b | 0.368 | < 0.001 | 0.525 | < 0.001 |  |
| PvMSP1-19 | 0.138 | < 0.001 | 0.377 | < 0.001 |  |
| Pvs16 | 0.268 | < 0.001 | 0.458 | < 0.001 |  |
| PvMSP8 | 0.214 | < 0.001 | 0.411 | < 0.001 |  |
| PvTRAg_28 | 0.231 | < 0.001 | 0.419 | < 0.001 |  |
| PvMSP3.10 | 0.220 | < 0.001 | 0.492 | < 0.001 |  |
| PvMSP7 | 0.121 | < 0.001 | 0.366 | < 0.001 |  |
