# Supplementary material for: Malaria transmission structure in the Peruvian Amazon through antibody signatures to Plasmodium vivax
Source: PLoS Negl Trop Dis. 2022 May 9;16(5):e0010415. doi: 10.1371/journal.pntd.0010415 (PMC9119515; doi:10.1371/journal.pntd.0010415)
Supplement: S3 Table — (DOCX) [file pntd.0010415.s008.docx]

| **S3 Table. Proportion of participants with *P. vivax* infections detected by microscopy, qPCR, and serology.** | | | | | |
| --- | --- | --- | --- | --- | --- |
| **Study site** | **Age group** | **N** | **LM (95% CI)** | **qPCR (95% CI)** | **SEM (95% CI)** |
| Iquitos | (0,5] | 56 | 0.05 (0.00-0.13) | 0.04 (0.00-0.09) | 0.07 (0.02-0.14) |
|  | (5,15] | 256 | 0.04 (0.02-0.06) | 0.05 (0.03-0.08) | 0.21 (0.16-0.27) |
|  | (15,30] | 199 | 0.03 (0.01-0.05) | 0.03 (0.01-0.06) | 0.36 (0.29-0.42) |
|  | (30,50] | 199 | 0.02 (0.00-0.04) | 0.08 (0.05-0.12) | 0.51 (0.44-0.58) |
|  | (50+] | 170 | 0.02 (0.01-0.05) | 0.06 (0.02-0.10) | 0.61 (0.53-0.68) |
| Mazán | (0,5] | 127 | 0.02 (0.00-0.06) | 0.04 (0.01-0.08) | 0.22 (0.15-0.29) |
|  | (5,15] | 347 | 0.01 (0.00-0.03) | 0.03 (0.01-0.04) | 0.34 (0.29-0.39) |
|  | (15,30] | 134 | 0.03 (0.01-0.06) | 0.04 (0.01-0.07) | 0.69 (0.60-0.76) |
|  | (30,50] | 218 | 0.02 (0.00-0.04) | 0.03 (0.01-0.05) | 0.79 (0.73-0.84) |
|  | (50+] | 198 | 0.01 (0.00-0.03) | 0.02 (0.00-0.03) | 0.85 (0.80-0.90) |
| N= Number of participants in each age group. LM: Light microscopy; SEM: Serological exposure marker. | | | | | |
