# Supplementary material for: Malaria transmission structure in the Peruvian Amazon through antibody signatures to Plasmodium vivax
Source: PLoS Negl Trop Dis. 2022 May 9;16(5):e0010415. doi: 10.1371/journal.pntd.0010415 (PMC9119515; doi:10.1371/journal.pntd.0010415)
Supplement: S4 Table — (DOCX) [file pntd.0010415.s009.docx]

| **S4 Table. Fixed Effects of multi-community multilevel models for seropositivity to *P. vivax.*** | | | | | | |
| --- | --- | --- | --- | --- | --- | --- |
|  | **Iquitos** | | | **Mazán** | | |
|  | **aOR** | **95% CI** | ***p*-value** | **aOR** | **95% CI** | ***p*-value** |
| **Null model** | | | | | | |
| Constant | 0.06 | (0.02-0.21) | <0.001 | 0.13 | (0.05-0.40) | <0.001 |
| **Age (Ref < 5 years)** | | | | | | |
| (5,15] years | 3.76 | (1.17-12.02) | 0.03 | 3.27 | (1.69-6.31) | <0.001 |
| (15,30] years | 11.35 | (3.35-38.45) | <0.001 | 13.68 | (5.78-32.38) | <0.001 |
| (30,50] years | 25.18 | (7.43-85.37) | <0.001 | 20.58 | (8.18-51.79) | <0.001 |
| (50 +] years | 28.51 | (8.46-96.05) | <0.001 | 28.34 | (11.05-72.68) | <0.001 |
| **Sex (Ref = Female)** | | | | | | |
| Male | 1.52 | (1.02-2.26) | 0.04 | 1.72 | (1.17-2.51) | 0.01 |
| **Education (Ref = None or Primary)** | | | | | | |
| Secondary school or higher | 0.60 | (0.40-0.91) | 0.02 | 0.85 | (0.51-1.41) | 0.53 |
| **Outdoor occupation (Ref = No)** | | | | | | |
| Yes | 0.93 | (0.47-1.84) | 0.84 | 2.51 | (1.43-4.41) | <0.001 |
| **Livestock inside dwelling (Ref = No)** | | | | | | |
| Yes | 1.06 | (0.66-1.72) | 0.80 | 0.59 | (0.35-0. 98) | 0.04 |
| **Fever symptom (Ref = No)** | | | | | | |
| Yes | 1.20 | (0.32-4.55) | 0.79 | 0.17 | (0.03-1.08) | 0.06 |
| **Housing type (Ref = Complete)** | | | | | | |
| Open house (0-3 walls) | 0.61 | (0.30-1.25) | 0.18 | 1.97 | (0.81-4.80) | 0.14 |
| **Travel last month (Ref = No)** | | | | | | |
| Yes | 1.61 | (0.42-6.13) | 0.49 | 1.22 | (0.80-1.87) | 0.35 |
| aOR: Adjusted Odds Ratio (aOR); 95% CI: 95 % Confidence interval. | | | | | | |
