# Supplementary material for: Malaria transmission structure in the Peruvian Amazon through antibody signatures to Plasmodium vivax
Source: PLoS Negl Trop Dis. 2022 May 9;16(5):e0010415. doi: 10.1371/journal.pntd.0010415 (PMC9119515; doi:10.1371/journal.pntd.0010415)
Supplement: S5 Table — (DOCX) [file pntd.0010415.s010.docx]

| **S5 Table. Random effects of multilevel logistic regression models of seropositivity to *P. vivax.*** | | | |
| --- | --- | --- | --- |
|  |  |  |  |
|  | **Var** | **ICC** | |
|  | **Est.** | **Est.** | **95% CI** |
| **Iquitos** | | | |
| household:community | 0.71 | 0.17 | (0.13-0.21) |
| community | 0.11 | 0.03 | (0.00-0.33) |
| **Mazán** | | | |
| household:community | 0.42 | 0.08 | (0.05-0.11) |
| community | 1.35 | 0.27 | (0.00-0.57) |
| Mixed-effects logistic models. Var: variance estimated by mixed-effects model; ICC: intra-class correlation coefficient; Est.: Standard deviation; 95% CI: 95 % Confidence interval. | | | |
|  |  |  |  |
