# Supplementary material for: Malaria transmission structure in the Peruvian Amazon through antibody signatures to Plasmodium vivax
Source: PLoS Negl Trop Dis. 2022 May 9;16(5):e0010415. doi: 10.1371/journal.pntd.0010415 (PMC9119515; doi:10.1371/journal.pntd.0010415)
Supplement: S6 Table — (DOCX) [file pntd.0010415.s011.docx]

| **S6 Table. Fixed Effects of multi-community multilevel models for *P. vivax* parasitaemia.** | | | | | | |
| --- | --- | --- | --- | --- | --- | --- |
|  | **Iquitos** | | | **Mazán** | | |
|  | **aOR** | **95% CI** | ***p*-value** | **aOR** | **95% CI** | ***p*-value** |
| **Null model** | | | | | | |
| Constant | 0.01 | (0.00-0.07) | <0.001 | 0.01 | (0.00-0.09) | <0.001 |
| **Age (Ref < 5 years)** | | | | | | |
| (5,15] years | 0.60 | (0.13-2.82) | 0.52 | 0.64 | (0.17-2.40) | 0.51 |
| (15,30] years | 0.38 | (0.06-2.36) | 0.30 | 0.41 | (0.06-2.79) | 0.37 |
| (30,50] years | 0.75 | (0.14-3.93) | 0.73 | 0.27 | (0.04-1.88) | 0.19 |
| (50 +] years | 0.65 | (0.11-3.74) | 0.63 | 0.13 | (0.02-1.07) | 0.06 |
| **Sex (Ref = Female)** | | | | | | |
| Male | 0.79 | (0.32-1.96) | 0. 61 | 1.57 | (0.63-3.90) | 0.33 |
| **Education (Ref = None or Primary)** | | | | | | |
| Secondary school or higher | 1.19 | (0.47-3.04) | 0.72 | 1.59 | (0.46-5.49) | 0.47 |
| **Outdoor occupation (Ref = No)** | | | | | | |
| Yes | 2.53 | (0.65-9.84) | 0.18 | 3.22 | (0.74-14.11) | 0.12 |
| **Livestock inside dwelling (Ref = No)** | | | | | | |
| Yes | 0.68 | (0.19-2.39) | 0.54 | 0.44 | (0.10-1.87) | 0.27 |
| **Housing type (Ref = Complete)** | | | | | | |
| Open house (0-3 walls) | 2.17 | (0.61-7.72) | 0.23 | 3.34 | (0.81-13.71) | 0. 09 |
| **Sprayed (Ref = No)** | | | | | | |
| Yes | 2.62 | (0.62-11.09) | 0.19 | 0.41 | (0.11-1.52) | 0.18 |
| aOR: Adjusted Odds Ratio (aOR); 95% CI: 95 % Confidence interval. | | | | | | |
