# Supplementary material for: Malaria transmission structure in the Peruvian Amazon through antibody signatures to Plasmodium vivax
Source: PLoS Negl Trop Dis. 2022 May 9;16(5):e0010415. doi: 10.1371/journal.pntd.0010415 (PMC9119515; doi:10.1371/journal.pntd.0010415)
Supplement: S7 Table — (DOCX) [file pntd.0010415.s012.docx]

| **S7 Table. Random effects of multilevel logistic regression models of *P. vivax* parasitaemia.** | | | |
| --- | --- | --- | --- |
|  |  |  |  |
|  | **Var** | **ICC** | |
|  | **Est.** | **Est.** | **95% CI** |
| **Iquitos** | | | |
| household:community | 70.22 | 0.94 | (0.14-0.91) |
| community | 1.12 | 0.02 | (0.00-0.33) |
| **Mazán** | | | |
| household:community | 1.56 | 0.25 | (0.20-0.30) |
| community | 1.28 | 0.21 | (0.00-0.57) |
| Mixed-effects logistic models. Var: variance estimated by mixed-effects model; ICC: intra-class correlation coefficient; Est.: Standard deviation; 95% CI: 95 % Confidence interval. | | | |
|  |  |  |  |
